# Supplementary material for: Mutations in PCYT2 disrupt etherlipid biosynthesis and cause a complex hereditary spastic paraplegia
Source: Brain. 2019 Oct 22;142(11):3382–97. doi: 10.1093/brain/awz291 (PMC6821184; doi:10.1093/brain/awz291)
Supplement: awz291_Supplementary_Data [file awz291_supplementary_data.zip › awz291-Suppl_data/Supplementary_Data4.zip!/Supplementary information 3 - fibroblast lipidomics.zip!/Fibroblast lipidomics/Patient1 vs Patient2/Statistical plots and data/DG species/Random forest.pdf]

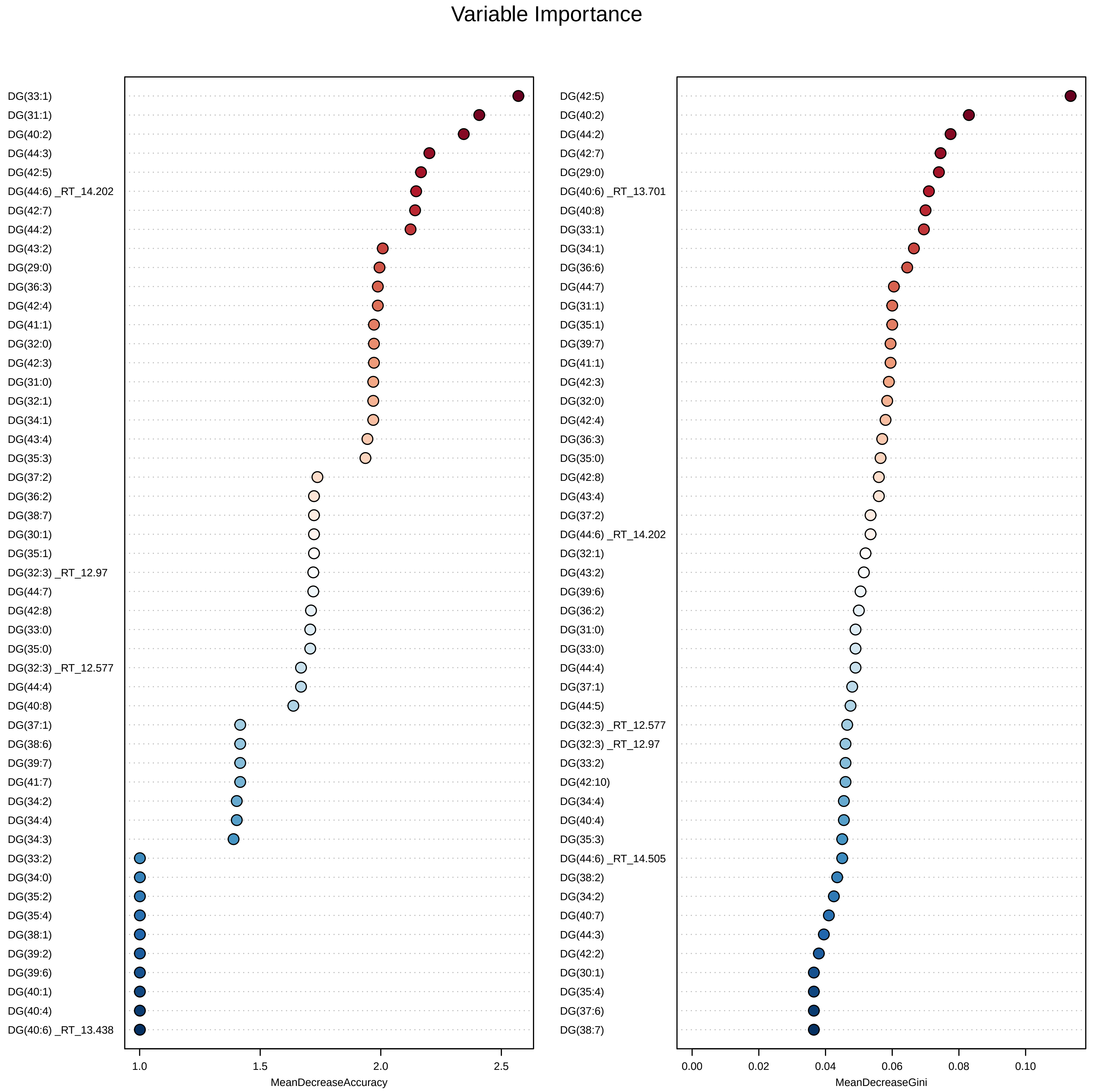
Variable Importance
DG(33:1)
DG(31:1)
DG(40:2)
DG(44:3)
DG(42:5)
DG(44:6) _RT_14.202
DG(42:7)
DG(44:2)
DG(43:2)
DG(29:0)
DG(36:3)
DG(42:4)
DG(41:1)
DG(32:0)
DG(42:3)
DG(31:0)
DG(32:1)
DG(34:1)
DG(43:4)
DG(35:3)
DG(37:2)
DG(36:2)
DG(38:7)
DG(30:1)
DG(35:1)
DG(32:3) _RT_12.97
DG(44:7)
DG(42:8)
DG(33:0)
DG(35:0)
DG(32:3) _RT_12.577
DG(44:4)
DG(40:8)
DG(37:1)
DG(38:6)
DG(39:7)
DG(41:7)
DG(34:2)
DG(34:4)
DG(34:3)
DG(33:2)
DG(34:0)
DG(35:2)
DG(35:4)
DG(38:1)
DG(39:2)
DG(39:6)
DG(40:1)
DG(40:4)
DG(40:6) _RT_13.438
1.0
1.5
2.0
2.5
MeanDecreaseAccuracy
DG(42:5)
DG(40:2)
DG(44:2)
DG(42:7)
DG(29:0)
DG(40:6) _RT_13.701
DG(40:8)
DG(33:1)
DG(34:1)
DG(36:6)
DG(44:7)
DG(31:1)
DG(35:1)
DG(39:7)
DG(41:1)
DG(42:3)
DG(32:0)
DG(42:4)
DG(36:3)
DG(35:0)
DG(42:8)
DG(43:4)
DG(37:2)
DG(44:6) _RT_14.202
DG(32:1)
DG(43:2)
DG(39:6)
DG(36:2)
DG(31:0)
DG(33:0)
DG(44:4)
DG(37:1)
DG(44:5)
DG(32:3) _RT_12.577
DG(32:3) _RT_12.97
DG(33:2)
DG(42:10)
DG(34:4)
DG(40:4)
DG(35:3)
DG(44:6) _RT_14.505
DG(38:2)
DG(34:2)
DG(40:7)
DG(44:3)
DG(42:2)
DG(30:1)
DG(35:4)
DG(37:6)
DG(38:7)
0.00
0.02
0.04
0.06
0.08
0.10
MeanDecreaseGini
